# Serological evidence of Yersinia pestis infection in rodents and carnivores in Northwestern Iran

**DOI:** 10.1371/journal.pntd.0011021

**Published:** 2023-01-20

**Authors:** Saber Esmaeili, Parisa Esmaeili, Ahmad Mahmoudi, Ahmad Ghasemi, Ali Mohammadi, Amin Bagheri, Aria Sohrabi, Farshid Rezaei, Hamed Hanifi, Amir Hesam Neamati, Mohammad Mehdi Gouya, Ehsan Mostafavi

**Affiliations:** 1 National Reference Laboratory for Plague, Tularemia and Q Fever, Research Centre for Emerging and Reemerging Infectious Diseases, Pasteur Institute of Iran, Akanlu, KabudarAhang, Hamadan, Iran; 2 Department of Epidemiology and Biostatics, Research Centre for Emerging and Reemerging Infectious Diseases, Pasteur Institute of Iran, Tehran, Iran; 3 Department of Biology, Faculty of Science, Urmia University, Urmia, Iran; 4 Reference Health Laboratories, Ministry of Health and Medical Education, Tehran, Iran; 5 Department of Medical Entomology and Vector Control, School of Public Health and National Institute of Health Research, Tehran University of Medical Sciences, Tehran, Iran; 6 Center for Communicable Disease Control, Ministry of Health and Medical Education, Tehran, Iran; University of Texas Medical Branch at Galveston, UNITED STATES

## Abstract

**Background:**

Plague may recur after several decades in its endemic regions; therefore, the continuous monitoring of wildlife is essential, even when no human cases are reported in the old foci. The present study was conducted to monitor rodents and their ectoparasites as well as carnivores to learn about the epidemiology of plague infection in an old focus of Iran.

**Methodology:**

The present study was conducted from 2019 to 2020 in Takestan county of Qazvin Province in northwestern Iran. Rodents were caught using live traps, and their fleas were separated. Blood and spleen specimens were taken from the captured rodents. Serum samples were also collected from sheepdogs and wild carnivores. The collected samples were tested by culture, serology (ELISA), and molecular methods to detect *Yersinia pestis* infection.

**Findings:**

A total of 399 small mammals were caught, of which 68.6% were *Meriones persicus*. A total of 2438 fleas were collected from the rodents, 95.3% of which were *Xenopsylla buxtoni*. Overall, 23 out of 377 tested rodents (5.7%, CI 95%, 3.9–9.0) had IgG antibodies against the *F1* antigen of *Y*. *pestis*, and all the positive samples belonged to *M*. *persicus*. Nine (4.8%) out of 186 collected sera from the sheepdogs’ serum and one serum from the *Canis aureus* had specific IgG antibodies against the *F1* antigen of *Y*. *pestis*. There were no positive cases of *Y*. *pestis* in the rodents and fleas based on the culture and real-time PCR.

**Conclusion:**

Serological evidence of *Y*. *pestis* circulation was observed in rodents and carnivores (sheepdogs and *C*. *aureus*). The presence of potential plague vectors and serological evidence of *Y*. *pestis* infection in the surveyed animals could probably raise the risk of infection and clinical cases of plague in the studied region. Training health personnel is therefore essential to encourage their detection of possible human cases of the disease.

## Author summary

Plague is a zoonotic disease caused by the gram-negative and rod-shaped *Yersinia pestis* bacterium. This disease has caused severe economic, social, and political crises throughout history. Rodents are known as the main plague reservoirs in nature and play a major role in the continued existence of enzootic cycles in natural foci. Nowadays, plague is a re-emerging disease that continues to be a serious health problem. Iran is regarded as an old endemic country for plague, from which several cases of epidemics have been reported throughout history. Given the recent findings in Iran and the re-emergence of plague in countries closer to Iran, the continuous surveillance of plague infection in wildlife seems necessary in different regions of the country. The present study was conducted to monitor rodents and their ectoparasites as well as carnivores to learn about the epidemiology of plague infection in old foci of Iran. Serological evidence of *Y*. *pestis* circulation was observed in rodents and carnivores (sheepdogs and *C. aureus*). The presence of potential plague vectors and serological evidence of *Y*. *pestis* infection in the surveyed animals could probably raise the risk of infection and clinical cases of plague in the studied region.

## Introduction

Plague is a zoonotic disease caused by the gram-negative and rod-shaped *Yersinia pestis* bacterium. This disease has caused severe economic, social, and political crises throughout history. Rodents are known as the main plague reservoirs in nature and play a major role in the continued existence of enzootic cycles in natural foci [1–3]. Fleas are the most important vectors of *Y*. *pestis*, and infected flea bites are the main transmitters of this disease to new hosts and humans. Human-to-human transmission of plague also occurs through both direct contact and inhalation of contaminated droplets in the case of pneumonic plague [4–6]. In humans, plague appears in three forms: Bubonic plague, septicemic plague, and pneumonic plague, all of which can occur separately or, rarely, at the same time. Bubonic plague is the most common form of plague with an incubation period of two to six days [7,8]. The mortality rates of septicemic and pneumonic forms have been reported as 30%-100% [9]. Another concern is the possibility of the cruel use of *Y*. *pestis* as biological weapon [10].

Despite the dramatic decline in plague cases in recent decades, sporadic cases have still been reported in endemic regions such as Asia, Africa, and America [4]. Nevertheless, epidemics and outbreaks occur occasionally, such that 2417 confirmed cases with 209 deaths were reported in Madagascar in 2017 [11].

Nowadays, plague is a re-emerging disease that continues to be a serious health problem. For instance, the plague re-emerged after 30 years in India in 1994 [12], after 95 years in Saudi Arabia in 1994 [13], after 40 years in Jordan in 1997 [14], after 50 years in Algeria in 2003 [15], and after 25 years in Libya in 2009 [16]. Iran is regarded as an old endemic country for plague, from which several cases of epidemics have been reported throughout history. Moreover, serological evidence of *Y*. *pestis* infection has been recently reported in rodents and dogs in western Iran [17].

Historical records indicate that two plague pandemics alongside several small epidemics have affected Iran throughout its history, and the plague has continued to have an active presence in Iran for centuries[18]. Human plague cases have been reported from different parts of the country in the past, and the last official report of a human plague dates back to Kurdistan Province (western Iran) in 1966 [19]. However, plague infection in wildlife has been identified in western and northwestern Iran, the region which is known as a natural old focus in the Middle East[18]. These countries (Afghanistan[20], Jordan[14], Saudi Arabia[13], Libya[21], India[22], Kyrgyzstan[23], Kazakhstan[24], close to Iran where the re-emergence of plague was recently reported.

Given the recent findings in Iran and the re-emergence of plague in countries closer to Iran, the continuous surveillance of plague infection in wildlife seems necessary in different regions of the country. For the last hundred years, the Pasteur Institute of Iran has been responsible for monitoring and diagnosing this disease in all regions of Iran. The present study was conducted in line with the constant monitoring program of the teams of the Research Center for Emerging and Re-emerging Infectious Diseases of the Pasteur Institute of Iran to clarify the current status of *Y*. *pestis* circulation in rodents and carnivores.

## Methods

### Ethics statement

All procedures for the collection and sampling of the animals were approved by the committees of Medical Ethics and Animal Work Ethics of Pasteur Institute of Iran (IR.PII.REC.1398.051).

### Study area

**Fig 1:**
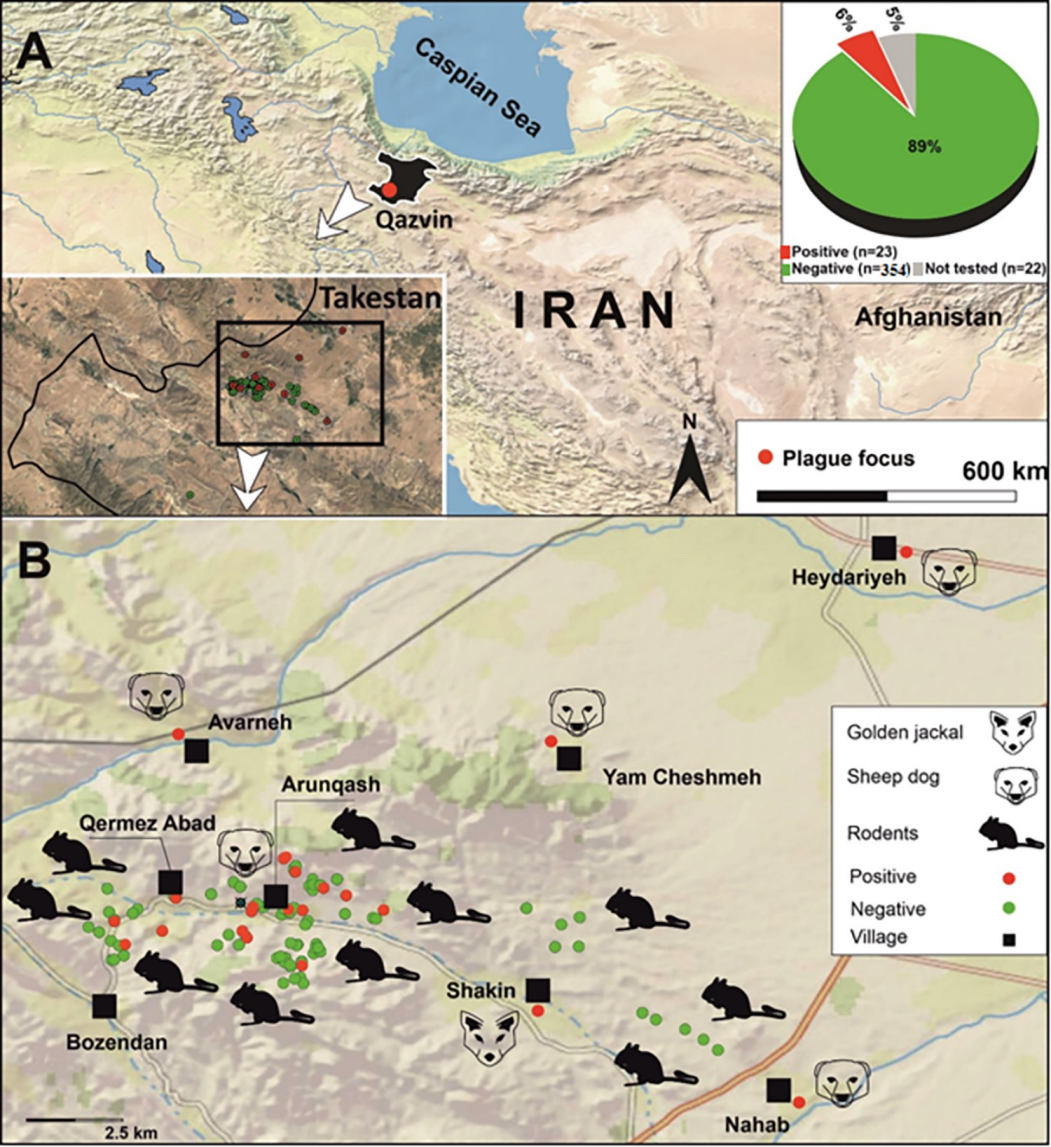
**Map of the study area in Iran, A) the geographic position of Qazvin Province, the pi-chart on the right-hand shows the proportion of the captured and positive rodents, B) sampling sites for rodents, and carnivores in Takestan county; red circles show serologically positive cases.** For both parts (A & B), used QGIS software, and the map layers were obtained from Natural Earth: https://www.naturalearthdata.com, all layers, and maps are public domain (http://www.naturalearthdata.com/about/terms-of-use/), and do not need permission for publication. The presented clip-arts in Fig 1 are drawn by authors using graphic design software (CorelDraw).

Takestan county, with an area of about 2536 Km^2^, is located 30 km away from the capital city of Qazvin in Qazvin province. The county is located at a longitude of 49° 10’ to 49° 48’ E of the Greenwich meridian and a latitude of 35° 40’ to 36° 21’ N of the equator. This county borders Buin Zahra County from the east, Avaj county from the south, Qazvin County from the north, and Zanjan province from the west (Fig 1). It consists of five cities and 133 villages. According to the latest official census of Iran, the population of this county is about 200,000.

In the present study, samples were collected from 60 different sites in 2019–2020 (for twenty days from 18 July to 07 August 2019, for ten days from October 14 to 23, 2019, and for 12 days from July 06 to 17, 2020).

### Small mammal and flea sampling

Small mammals were captured using live wooden traps baited by dates or cucumbers. Small mammals’ ectoparasites were isolated in the field and kept in a normal saline solution. The small mammals were then sent to the laboratory with their isolated fleas. The small mammals were identified based on morphological characteristics using the available identification keys [25,26]. The collected fleas were identified based on the available identification keys as well [27].

In the laboratory, after anaesthesia by using Isoflurane, blood samples were taken from each small mammal, and serum samples were extracted. Sera samples were stored at -20°C. Afterwards, under aseptic conditions, the spleen tissue of small mammals was removed and divided into two parts and placed inside two separate sterile microtubes (for culture and molecular examination). Spleen samples were stored at 4°C until it was time for the culture.

### Sheepdog and wild carnivore sampling

Upon coordination with the Veterinary Organization, blood samples were taken from sheepdogs after obtaining the consent of their owners. Ten ml of blood was collected from each dog, and the blood samples were centrifuged at 1500 rpm for 15 min to isolate their sera. After obtaining the necessary hunting licenses, blood samples were taken from the hunted wild carnivores, and their sera were extracted and kept at -20°C until the analysis.

### Culture of *Y*. *pestis*

Spleen and flea samples were homogenized in normal saline under sterile conditions, and their suspension was obtained. Then, 50 μl of the suspension was cultured in MacConkey agar and incubated at 28°C for 72 h. The microbial cultures were examined daily, and the suspect colonies grown were examined using gram staining and biochemical tests, including oxidase, catalase, lactose fermentation, motility, and indole tests. Finally, the suspect isolates for *Y*. *pestis* were examined by Real-time PCR.

### Molecular detection of *Y*. *pestis*

Genomic DNA was extracted from the spleen specimens of small mammals and the collected fleas using a commercial kit (Viragene DNA Isolation Kit, Iran). Furthermore, the DNA of the suspect isolates for *Y*. *pestis* in bacterial culture were also extracted by boiling method. All the extracted DNA samples were kept at -20°C until the analysis.

All the samples were tested for plasmid (pMT1 [*caf1*], pPCP1 [*pla]*) and chromosomal (yihN) genes of *Y*. *pestis* (Table 1) using multiplex Real-Time PCR [28].

**Table 1:** Primers and probes were used to detect *Y*. *pestis*.

Target	Primer/Probe	Sequences (5’ to 3’)	Amplicon Size (bp)
*yihN* (chromosome)	Forward Reverse Probe	CGCTTTACCTTCACCAAACTGAAC GGTTGCTGGGAACCAAAGAAGA Cy5-TAAGTACATCAATCACACCGCGACCCGCTT-BHQ2	128
*Caf1* (pMT1)	Forward Reverse Probe	CCGTTATCGCCATTGCATTATTTGG GCCAAGAGTAAGCGTACCAACAAG Hex-AAGCACCACTGCAACGGCAACTCTT-BHQ1	194
*pla* (pPCP1)	Forward Reverse Probe	ATTGGACTTGCAGGCCAGTATC ATAACGTGAGCCGGATGTCTTC FAM-AAATTCAGCGACTGGGTTCGGGCACA-BHQ1	144

### *Y*. *pestis* serology

The sera samples collected from small mammals and dogs were tested to detect IgG antibodies against the *F1* antigen of *Y*. *pestis* using an ELISA test. F1 antigen and positive negative control sera for rodents and dogs were provided by the Institut Pasteur in Madagascar (World Health Organization Collaborating Centre for Plague). Also, The ELISA test was performed according to the ELISA protocol recommended by the Plague unit of Institut Pasteur in Madagascar with a little modification [29]. Briefly, for the IgG-anti F1 antigen, 96 well microtiter ELISA plates (PolySorp, Nunc, Germany) were coated with purified F1 antigen of *Y*. *pestis* in l in carbonate buffer, pH 9.6, at 4°C overnight (3.8 μg/ml, 70 μl per well). Plates were washed three times using a washing solution (phosphate-buffered saline (PBS)/ 0.1% Tween 20%), then incubated with blocking buffer (PBS/ 0.1% Tween 20 Tween 20% with 5% skim milk (Sigma-Aldrich, Switzerland)) at 37°C for 2 h. After three times washing of plates using wash solution, all sera samples were diluted 1/100 in PBS/ 0.1% Tween 20 Tween 20% with 5% skim milk, transferred to the plate (100 μl/well), and incubated at 37°C for 1 h. Plates were washed again three times, and anti-specific IgG horseradish peroxidase conjugate (rat (1/7000), dogs (1/15000), rabbit (1/10000)) (Abcam, USA) diluted in PBS/ 0.1% Tween 20% with 5% skim milk, and then 100 μl/well of them had added to the ELISA plates based on tested animals. The plates were incubated at 37°C for 1 h. After the final washing steps (four times washing), 100 μl tetramethylbenzidine/peroxide (TMB, Seramun, Germany) per well was added as substrate and incubated for 30 min at room temperature. Finally, 100 μl stop solution (H_2_SO_4_) was added for all wells and the results were determined by measuring absorbance at 450 nm with an automated microplate reader (BioTek- USA). In each experiment, a substrate control (blank), negative control, and positive control were run in parallel with the test samples according to the type of animal tested. The ELISA protocol was added to the manuscript. Also, the threshold (cut-off point) of positivity for rodents and carnivores was set at 0.35 and 0.450 (based on ELISA protocol and our previous studies), respectively, and if the optical density of the sample were above the defined threshold, considered positive [17].

## Results

### Identification of small mammals

During the two years of the field survey, a total of 399 small mammals were captured, including 191 (47.87%) collected in 2019 and 208 (52.13%) in 2020 (S1 Dataset). In this study, 68.7% of the rodents were belonging to the Persian jird (*Meriones persicus*). The other prevalent species collected were *Microtus qazvinensis* (23.8%) and *Meriones vinogradovi* (1.5%) (Table 2).

**Table 2:** Scientific name and number of the small mammals surveyed in the present study from Takestan county, Qazvin province in 2019–2020.

Species	No.			
	2019		2020	Total
	July	October	July	
***Meriones persicus***	120	24	130	274
***Microtus qazvinensis***	24	6	65	95
***Mus musculus***	1	0	1	2
***Apodemus witherbyi***	2	2	2	6
***Meriones vinogradovi***	4	0	2	6
***Nothocricetulus migratorius***	2	0	0	2
***Arvicola persicus***	0	2	4	6
***Scarturus indicus***	2	0	0	2
***Scarturus williamsi***	0	0	3	3
***Crocidura suaveolens***	0	1	1	2
***Meriones tristrami***	0	1	0	1
**Total**	155	36	208	399

### Identification of fleas

In the present study, a total of 2438 fleas were isolated from the captured rodents, revealing a load of 6.1 fleas per rodent. In the first year of the study, the fleas were not subjected to species identification (n = 721, 29.6%), and were directly used for the detection of Y. pestis by culture and molecular methods. In the second year of the study, 95.3% of the collected fleas (n = 1717) were identified as *Xenopsylla buxtoni*, which were collected from four rodent species: *M*. *persicus* (86%), *M*. *qazvinensis* (13%), *M*. *vinogradovi* (0.7%) and *Apodemus witherbyi* (0.3%). Furthermore, 3.4% of the collected fleas were *Nosopsyllus mikulini*, which were collected from *M*. *persicus* (52.5%), and *M*. *qazvinensis* (47.5%). Besides, a few *Ctenophthalmus rettigi smiti* (1.2%) were identified only from *M*. *persicus*.

### Sera samples of carnivores

A total of 186 blood samples (S1 Dataset) were collected from sheepdogs in the studied area during the first year (2019). Moreover, three samples from golden jackal (*Canis aureus*) and two samples from red fox (*Vulpes vulpes*) were captured.

### *Y*. *pestis* culture

None of the 399 spleen samples from rodents was infected by *Y*. *pestis* using the culture method. The microbial culture of fleas was likely negative for *Y*. *pestis*.

### Molecular tracing of plague

Neither small mammals nor fleas tested positive for the plague infection by Real-Time PCR.

### *Y*. *pestis* serology

Unfortunately, we could not obtain sera samples from 22 small mammals, and only 377 sera of small mammals were used for serological investigation. Twenty-three (6.1%, CI 95%: 3.6–8.3) out of 377 rodents had IgG antibodies against the *F1* antigen of *Y*. *pestis* and all the positive samples were belonging to *M*. *persicus*. Also, 23 (8.7%) of 263 tested *M*. *persicus* were serologically positive for plague in this study.

Furthermore, nine (4.8%) out of 186 sera obtained from sheepdogs had specific IgG antibodies against the *F1* antigen of *Y*. *pestis*. In addition, one serum collected from golden jackal had a specific IgG antibody against the F1 antigen of *Y*. *pestis*. There were no positive serological cases in the fox samples.

## Discussion

There was serological evidence of *Y*. *pestis* circulation in rodents and carnivores in our study area. *Y*. *pestis* can survive in natural foci in several ways, such as in symbiosis with soil protozoa, within the body of deceased fleas and rodents, and in resistance reservoirs[1]. During silent periods, no human clinical cases may be observed for several years, or sporadic clinical cases may occur that can be treated with antibiotics without clinical or laboratory diagnosis [30–32]. In plague-endemic areas like Iran, human cases of plague can reemerge after decades; therefore, constant monitoring is required even if no human cases are reported in the old foci.

*M*. *persicus* was the most dominant rodent (68.6%) caught in the present study. Based on serological results, 8.7% of *M*. *persicus*, 23 had IgG against the *F1* antigen of *Y*. *pestis*. Plague reservoirs vary from one region to another [1], and four species of the genus *Meriones* have been assumed to be the main reservoirs in Iranian natural plague foci: *M*. *persicus*, *M*. *libycus*, *M*. *tristrami*, and *M*. *vinogradovi*. *M*. *persicus* and *M*. *libycus* are considered relatively resistant, but the two latter are known as susceptible species [33, 34]. In an earlier report in 2013, after several years of serological study of plague in an old historical focus of Iran (about 140 km to the west of our study area), 4.2% of *M*. *persicus* had IgG against the *F1* antigen of *Y*. *pestis* [17]. Even though the molecular detection and culture of *Y*. *pestis* were not successful in the present study or recent monitoring studies in the old foci, the presence of positive serological cases in rodents indicates previous exposure to *Y*. *pestis*. Therefore, it is not unreasonable to see positive molecular cases as well as *Y*. *pestis* cultures in future studies in old foci.

Based on the results of the present study, there was a 4.8% positive serum prevalence of IgG against the *F1* antigen of *Y*. *pestis* in sheepdogs, which is higher than that of our previous study (3.4%) in an old focus [17]. Carnivores are crucial animals for epidemiological studies in plague monitoring. *Y*. *pestis* infection in dogs is usually asymptomatic, or causes only mild self-limiting fever, and these animals have acceptable levels of antibody to the *F1* antigen, which is more stable than rodents [35]. Dogs can play a role in human infection by transmitting rodent fleas to human habitats [36,37]. There are very rare cases of direct transmission of plague from dogs to humans (without the intervention of arthropod vectors) [38].

In the present study, the serum sample of a golden jackal contained IgG against the *F1* antigen of *Y*. *pestis*, which confirmed the circulation of plague in wildlife in the studied region. Although symptomatic diseases are rare in wild and domestic carnivores such as jackals and dogs, the possibility of *Y*. *pestis* infection should be considered in infected dogs by their exposure to wildlife reservoirs in areas where the infection is endemic [39]. Rodents are food sources of carnivores such as golden jackals and red foxes; the constant contact between these mammals is the reason why it is crucial to investigate carnivores as part of monitoring studies in natural foci. Therefore, sampling of carnivores in native areas can be used as an early warning system to find strong evidence of plague circulation, which is very important when human plague cases occur normally [40]. The results of the present study provide evidence that *Y*. *pestis* is still present in Iran and has active circulation among its reservoirs. Constant monitoring is needed in these areas, even in quiescent period with no human cases. Therefore, the timely detection of plague by the *F1* antigen test in dogs in the present study is valuable for the effective clinical management of plague cases and timely response by way of health interventions. Serological studies of plague in sheepdogs can help to elucidate the duration of presence of F1 antibodies in dogs.

Fleas are the main vectors of plague. *Xenopsylla* is known as the main vector of plague in Iran [41]. *X*. *astia*, *X*. *baxtoni*, and *X*. *cheopis* are the main species of *Xenopsylla* that are involved in the transmission of *Y*. *pestis* [42]. In the present study, about 95.3% of the fleas collected from rodents were from *X*. *buxtoni* species. In addition, *Y*. *pestis* infection was detected in our research using the serological method in rodents and dogs, which indicates a bacterial cycle in rodents; for this reason, we should expect infections and clinical cases of plague in these old foci, which continue to have potential vectors for the transmission of plague and has shown a serological trace of *Y*. *pestis*. To overcome possible outbreaks of plague, health personnel must be informed about the continued presence of this disease and receive the necessary training.

The occurrence of plague epidemics in human communities could be managed through the monitoring and control of potential and major reservoirs and vectors such as rodents, carnivores, and fleas in endemic areas, as well as through the rapid detection and treatment of cases. Therefore, constant monitoring of wildlife has to be a critical aspect of the surveillance systems in endemic regions for the plague. Otherwise, widespread rodent proliferation and silent outbreaks of plague in human communities can be expected [43]. To elucidate the main roles of rodents, carnivores, and their fleas in the plague transmission cycle, more studies are needed on old and known foci in a wide geographical area.

## Supporting information

S1 Dataset The data of sampling from small mammals and carnivores in this study. (XLSX)
